# Supplementary material for: Representation from India in multinational, interventional, phase 2 or 3 trials registered in Clinical Trials Registry-India: A cross-sectional study
Source: PLoS One. 2023 Sep 20;18(9):e0284434. doi: 10.1371/journal.pone.0284434 (PMC10511072; doi:10.1371/journal.pone.0284434)
Supplement: S1 File — (DOC) [file pone.0284434.s001.doc]

**S1 File: CTRI numbers of sample trials registered with the Indian registry CTRI, and the URLs at which they are available.**

1. **CTRI/2014/07/004722**

<https://ctri.nic.in/Clinicaltrials/pmaindet2.php?trialid=8924&EncHid=&userName=CTRI/2014/07/004722>

1. **CTRI/2020/06/026192**

<https://ctri.nic.in/Clinicaltrials/pmaindet2.php?trialid=44815&EncHid=&userName=CTRI/2020/06/026192>

1. **CTRI/2019/10/021535**

<https://ctri.nic.in/Clinicaltrials/pmaindet2.php?trialid=35181&EncHid=&userName=CTRI/2019/10/021535>

1. **CTRI/2018/06/014543**

<https://ctri.nic.in/Clinicaltrials/pmaindet2.php?trialid=23591&EncHid=&userName=CTRI/2018/06/014543>

1. **CTRI/2020/07/026641**

<https://ctri.nic.in/Clinicaltrials/pmaindet2.php?trialid=43746&EncHid=&userName=CTRI/2020/07/026641>
