# Supplementary material for: Representation from India in multinational, interventional, phase 2 or 3 trials registered in Clinical Trials Registry-India: A cross-sectional study
Source: PLoS One. 2023 Sep 20;18(9):e0284434. doi: 10.1371/journal.pone.0284434 (PMC10511072; doi:10.1371/journal.pone.0284434)
Supplement: S2 File — (DOCX) [file pone.0284434.s002.docx]

**S2 File. The R script used to download data from CTRI, process them and store them in an SQLite database.**

S2 File is available at <https://osf.io/bsncw>
