# Supplementary material for: Representation from India in multinational, interventional, phase 2 or 3 trials registered in Clinical Trials Registry-India: A cross-sectional study
Source: PLoS One. 2023 Sep 20;18(9):e0284434. doi: 10.1371/journal.pone.0284434 (PMC10511072; doi:10.1371/journal.pone.0284434)
Supplement: S3 File — (DOCX) [file pone.0284434.s003.docx]

**S3 File. Details of 39,821 records from CTRI, stored in an SQLite database.**

S3 File is available at <https://osf.io/kr9f8>
