# Supplementary material for: Representation from India in multinational, interventional, phase 2 or 3 trials registered in Clinical Trials Registry-India: A cross-sectional study
Source: PLoS One. 2023 Sep 20;18(9):e0284434. doi: 10.1371/journal.pone.0284434 (PMC10511072; doi:10.1371/journal.pone.0284434)
Supplement: S4 File — (DOCX) [file pone.0284434.s004.docx]

**S4 File. The schema of the SQLite database.***

| **Database schema**  **Tables (27)** |  |  |
| --- | --- | --- |
| **Name** | **Type** | **Schema** |
| **Contact_person_public_query** |  | CREATE TABLE "Contact_person_public_query" ("Trial_ID" INTEGER, "CTRI_Number" TEXT, "Name" TEXT, "Designation" TEXT, "Affiliation" TEXT, "Address" TEXT, "Phone" TEXT, "Fax" TEXT, "Email" TEXT) |
| Trial_ID | INTEGER | "Trial_ID" INTEGER |
| CTRI_Number | TEXT | "CTRI_Number" TEXT |
| Name | TEXT | "Name" TEXT |
| Designation | TEXT | "Designation" TEXT |
| Affiliation | TEXT | "Affiliation" TEXT |
| Address | TEXT | "Address" TEXT |
| Phone | TEXT | "Phone" TEXT |
| Fax | TEXT | "Fax" TEXT |
| Email | TEXT | "Email" TEXT |
| **Contact_person_scientific_query** |  | CREATE TABLE "Contact_person_scientific_query" ("Trial_ID" INTEGER, "CTRI_Number" TEXT, "Name" TEXT, "Designation" TEXT, "Affiliation" TEXT, "Address" TEXT, "Phone" TEXT, "Fax" TEXT, "Email" TEXT) |
| Trial_ID | INTEGER | "Trial_ID" INTEGER |
| CTRI_Number | TEXT | "CTRI_Number" TEXT |
| Name | TEXT | "Name" TEXT |
| Designation | TEXT | "Designation" TEXT |
| Affiliation | TEXT | "Affiliation" TEXT |
| Address | TEXT | "Address" TEXT |
| Phone | TEXT | "Phone" TEXT |
| Fax | TEXT | "Fax" TEXT |
| Email | TEXT | "Email" TEXT |
| **Countries for recruitment** |  | CREATE TABLE "Countries for recruitment" ("Trial ID" INTEGER, "CTRI Number" TEXT, "Countries" TEXT) |
| Trial ID | INTEGER | "Trial ID" INTEGER |
| CTRI Number | TEXT | "CTRI Number" TEXT |
| Countries | TEXT | "Countries" TEXT |
| **DCGI status** |  | CREATE TABLE "DCGI status" ("Trial ID" INTEGER, "CTRI Number" TEXT, "DCGI status" TEXT) |
| Trial ID | INTEGER | "Trial ID" INTEGER |
| CTRI Number | TEXT | "CTRI Number" TEXT |
| DCGI status | TEXT | "DCGI status" TEXT |
| **Dates table** |  | CREATE TABLE "Dates table" ("Trial_ID" INTEGER, "CTRI_Number" TEXT, "Last_modified_on" TEXT, "Date_first_enrollment_India" TEXT, "Date_first_enrollment_GLobal" TEXT, "Date_of_study_completion_India" TEXT, "Date_of_study_completion_Global” TEXT) |
| Trial_ID | INTEGER | "Trial_ID" INTEGER |
| CTRI_Number | TEXT | "CTRI_Number" TEXT |
| Last_modified_on | TEXT | "Last_modified_on" TEXT |
| Date_first_enrollment_India | TEXT | "Date_first_enrollment_India" TEXT |
| Date_first_enrollment_GLobal | TEXT | "Date_first_enrollment_GLobal" TEXT |
| Date_of_study_completion_India | TEXT | "Date_of_study_completion_India" TEXT |
| Date_of_study_completion_Global | TEXT | "Date_of_study_completion_Global" TEXT |
| **Estimated_trial_duration** |  | CREATE TABLE "Estimated_trial_duration" ("Trial_ID" INTEGER, "CTRI_Number" TEXT, "Estimated_trial_duration" TEXT) |
| Trial_ID | INTEGER | "Trial_ID" INTEGER |
| CTRI_Number | TEXT | "CTRI_Number" TEXT |
| Estimated_trial_duration | TEXT | "Estimated_trial_duration" TEXT |
| **Ethics_committee** |  | CREATE TABLE "Ethics_committee" ("Trial_ID" INTEGER, "CTRI_Number" TEXT, "No_of_ECs" INTEGER, "Name_of_Committee" TEXT, "Approval_Status" TEXT) |
| Trial_ID | INTEGER | "Trial_ID" INTEGER |
| CTRI_Number | TEXT | "CTRI_Number" TEXT |
| No_of_ECs | INTEGER | "No_of_ECs" INTEGER |
| Name_of_Committee | TEXT | "Name_of_Committee" TEXT |
| Approval_Status | TEXT | "Approval_Status" TEXT |
| **Exclusion_criteria** |  | CREATE TABLE "Exclusion_criteria" ("Trial_ID" INTEGER, "CTRI_Number" TEXT, "Exclusion_details" TEXT) |
| Trial_ID | INTEGER | "Trial_ID" INTEGER |
| CTRI_Number | TEXT | "CTRI_Number" TEXT |
| Exclusion_details | TEXT | "Exclusion_details" TEXT |
| **Health_conditions** |  | CREATE TABLE "Health_conditions" ("Trial_ID" INTEGER, "CTRI_Number" TEXT, "Health_Type" TEXT, "COndition" TEXT) |
| Trial_ID | INTEGER | "Trial_ID" INTEGER |
| CTRI_Number | TEXT | "CTRI_Number" TEXT |
| Health_Type | TEXT | "Health_Type" TEXT |
| COndition | TEXT | "COndition" TEXT |
| **Inclusion_criteria** |  | CREATE TABLE "Inclusion_criteria" ("Trial_ID" INTEGER, "CTRI_Number" TEXT, "Age_From" TEXT, "Age_To" TEXT, "Gender" TEXT, "Details" TEXT) |
| Trial_ID | INTEGER | "Trial_ID" INTEGER |
| CTRI_Number | TEXT | "CTRI_Number" TEXT |
| Age_From | TEXT | "Age_From" TEXT |
| Age_To | TEXT | "Age_To" TEXT |
| Gender | TEXT | "Gender" TEXT |
| Details | TEXT | "Details" TEXT |
| **Intervention_table** |  | CREATE TABLE "Intervention_table" ("Trial_ID" INTEGER, "CTRI_Number" TEXT, "Intervention_Name" TEXT, "Intervention_details" TEXT, "Comparator_Name" TEXT, "Comparator_details" TEXT) |
| Trial_ID | INTEGER | "Trial_ID" INTEGER |
| CTRI_Number | TEXT | "CTRI_Number" TEXT |
| Intervention_Name | TEXT | "Intervention_Name" TEXT |
| Intervention_details | TEXT | "Intervention_details" TEXT |
| Comparator_Name | TEXT | "Comparator_Name" TEXT |
| Comparator_details | TEXT | "Comparator_details" TEXT |
| **Method_table** |  | CREATE TABLE "Method_table" ("Trial_ID" INTEGER, "CTRI_Number" TEXT, "Method_of_Generating_Random_Sequence" TEXT, "Method_of_Concealment" TEXT, "Blinding_or_Masking" TEXT) |
| Trial_ID | INTEGER | "Trial_ID" INTEGER |
| CTRI_Number | TEXT | "CTRI_Number" TEXT |
| Method_of_Generating_Random_Sequence | TEXT | "Method_of_Generating_Random_Sequence" TEXT |
| Method_of_Concealment | TEXT | "Method_of_Concealment" TEXT |
| Blinding_or_Masking | TEXT | "Blinding_or_Masking" TEXT |
| **Primary_outcomes** |  | CREATE TABLE "Primary_outcomes" ("Trial_ID" INTEGER, "CTRI_Number" TEXT, "Primary_Outcome" TEXT, "primary_Outcome_timepoints" TEXT) |
| Trial_ID | INTEGER | "Trial_ID" INTEGER |
| CTRI_Number | TEXT | "CTRI_Number" TEXT |
| Primary_Outcome | TEXT | "primary_Outcome" TEXT |
| primary_Outcome_timepoints | TEXT | "primary_Outcome_timepoints" TEXT |
| **Primary_sponsor** |  | CREATE TABLE "Primary_sponsor" ("Trial_ID" INTEGER, "CTRI_Number" TEXT, "primary_sponsor_name" TEXT, "primary_sponsor_address" TEXT, "Type_of_Sponsor" TEXT) |
| Trial_ID | INTEGER | "Trial_ID" INTEGER |
| CTRI_Number | TEXT | "CTRI_Number" TEXT |
| primary_sponsor_name | TEXT | "primary_sponsor_name" TEXT |
| primary_sponsor_address | TEXT | "primary_sponsor_address" TEXT |
| Type_of_Sponsor | TEXT | "Type_of_Sponsor" TEXT |
| **Principal_investigator** |  | CREATE TABLE "Principal_investigator" ("Trial_ID" INTEGER, "CTRI_Number" TEXT, "Name" TEXT, "Designation" TEXT, "Affiliation" TEXT, "Address" TEXT, "Phone" TEXT, "Fax" TEXT, "Email" TEXT) |
| Trial_ID | INTEGER | "Trial_ID" INTEGER |
| CTRI_Number | TEXT | "CTRI_Number" TEXT |
| Name | TEXT | "Name" TEXT |
| Designation | TEXT | "Designation" TEXT |
| Affiliation | TEXT | "Affiliation" TEXT |
| Address | TEXT | "Address" TEXT |
| Phone | TEXT | "Phone" TEXT |
| Fax | TEXT | "Fax" TEXT |
| Email | TEXT | "Email" TEXT |
| **Publication_details** |  | CREATE TABLE "Publication_details" ("Trial_ID" INTEGER, "CTRI_Number" TEXT, "Publication_Details" TEXT) |
| Trial_ID | INTEGER | "Trial_ID" INTEGER |
| CTRI_Number | TEXT | "CTRI_Number" TEXT |
| Publication_Details | TEXT | "Publication_Details" TEXT |
| **Recruitment_details** |  | CREATE TABLE "Recruitment_details" ("Trial_ID" INTEGER, "CTRI_Number" TEXT, "Recruitment_Status_Global” TEXT, "Recruitment_Status_India" TEXT) |
| Trial_ID | INTEGER | "Trial_ID" INTEGER |
| CTRI_Number | TEXT | "CTRI_Number" TEXT |
| Recruitment_Status_Global | TEXT | "Recruitment_Status_Global" TEXT |
| Recruitment_Status_India | TEXT | "Recruitment_Status_India" TEXT |
| **Registration_details** |  | CREATE TABLE "Registration_details" ("Trial_ID" INTEGER, "CTRI_Number" TEXT, "Registered_on" TEXT, "Registration_type" TEXT) |
| Trial_ID | INTEGER | "Trial_ID" INTEGER |
| CTRI_Number | TEXT | "CTRI_Number" TEXT |
| Registered_on | TEXT | "Registered_on" TEXT |
| Registration_type | TEXT | "Registration_type" TEXT |
| **Secondary_id** |  | CREATE TABLE "Secondary_id" ("Trial_ID" INTEGER, "CTRI_Number" TEXT, "Secondary_ID" TEXT, "Identifier" TEXT) |
| Trial_ID | INTEGER | "Trial_ID" INTEGER |
| CTRI_Number | TEXT | "CTRI_Number" TEXT |
| Secondary_ID | TEXT | "Secondary_ID" TEXT |
| Identifier | TEXT | "Identifier" TEXT |
| **Secondary_outcomes** |  | CREATE TABLE "Secondary_outcomes" ("Trial_ID" INTEGER, "CTRI_Number" TEXT, "Secondary_Outcome" TEXT, "Secondary_Outcome_timepoints" TEXT) |
| Trial_ID | INTEGER | "Trial_ID" INTEGER |
| CTRI_Number | TEXT | "CTRI_Number" TEXT |
| Secondary_Outcome | TEXT | "Secondary_Outcome" TEXT |
| Secondary_Outcome_timepoints | TEXT | "Secondary_Outcome_timepoints" TEXT |
| **Secondary_sponsor** |  | CREATE TABLE "Secondary_sponsor" ("Trial_ID" INTEGER, "CTRI_Number" TEXT, "Secondary_Outcome" TEXT, "Secondary_Outcome_timepoints" TEXT) |
| Trial_ID | INTEGER | "Trial_ID" INTEGER |
| CTRI_Number | TEXT | "CTRI_Number" TEXT |
| Secondary_Outcome | TEXT | "Secondary_Outcome" TEXT |
| Secondary_Outcome_timepoints | TEXT | "Secondary_Outcome_timepoints" TEXT |
| **Sites_of_study** |  | CREATE TABLE "Sites_of_study" ("Trial_ID" INTEGER, "CTRI_Number" TEXT, "No_of_Sites" TEXT, "Name_of_Prinicpal_Investigator" TEXT, “Site_Name" TEXT, "Site_Address" TEXT, "Other_details" TEXT) |
| Trial_ID | INTEGER | "Trial_ID" INTEGER |
| CTRI_Number | TEXT | "CTRI_Number" TEXT |
| No_of_Sites | TEXT | "No_of_Sites" TEXT |
| Name_of_Prinicpal_Investigator | TEXT | "Name_of_Prinicpal_Investigator" TEXT |
| Site_Name | TEXT | "Site_Name" TEXT |
| Site_Address | TEXT | "Site_Address" TEXT |
| Other_details | TEXT | "Other_details" TEXT |
| **Source_of_Monetary_or_Material_Support** |  | CREATE TABLE "Source_of_Monetary_or_Material_Support" ("Trial_ID" INTEGER, "CTRI_Number" TEXT, "Source_of_Monetary_or_Material_Support” TEXT) |
| Trial_ID | INTEGER | "Trial_ID" INTEGER |
| CTRI_Number | TEXT | "CTRI_Number" TEXT |
| Source_of_Monetary_or_Material_Support | TEXT | "Source_of_Monetary_or_Material_Support" TEXT |
| **Study_details** |  | CREATE TABLE "Study_details" ("Trial_ID" INTEGER, "CTRI_Number" TEXT, "Type_of_Trial" TEXT, "Type_of_Study" TEXT, "Study_design" TEXT, "Phase" TEXT, "Trial_Acronym" TEXT, "Post_graduation_thesis" TEXT) |
| Trial_ID | INTEGER | "Trial_ID" INTEGER |
| CTRI_Number | TEXT | "CTRI_Number" TEXT |
| Type_of_Trial | TEXT | "Type_of_Trial" TEXT |
| Type_of_Study | TEXT | "Type_of_Study" TEXT |
| Study_design | TEXT | "Study_design" TEXT |
| Phase | TEXT | "Phase" TEXT |
| Trial_Acronym | TEXT | "Trial_Acronym" TEXT |
| Post_graduation_thesis | TEXT | "Post_graduation_thesis" TEXT |
| **Study_summary** |  | CREATE TABLE "Study_summary" ("Trial_ID" INTEGER, "CTRI_Number" TEXT, "Brief_Summary" TEXT) |
| Trial_ID | INTEGER | "Trial_ID" INTEGER |
| CTRI_Number | TEXT | "CTRI_Number" TEXT |
| Brief_Summary | TEXT | "Brief_Summary" TEXT |
| **Study_titles** |  | CREATE TABLE "Study_titles" ("Trial_ID" INTEGER, "CTRI_Number" TEXT, "Public_Title" TEXT, "Scientific_Title" TEXT) |
| Trial_ID | INTEGER | "Trial_ID" INTEGER |
| CTRI_Number | TEXT | "CTRI_Number" TEXT |
| Public_Title | TEXT | "Public_Title" TEXT |
| Scientific_Title | TEXT | "Scientific_Title" TEXT |
| **Target_sample_size** |  | CREATE TABLE "Target_sample_size" ("Trial_ID" INTEGER, "CTRI_Number" TEXT, "sample_size" TEXT) |
| Trial_ID | INTEGER | "Trial_ID" INTEGER |
| CTRI_Number | TEXT | "CTRI_Number" TEXT |
| sample_size | TEXT | "sample_size" TEXT |

* This file is broadly similar to that used for various other projects of our group, whose manuscripts are being processed.
