# Supplementary material for: Representation from India in multinational, interventional, phase 2 or 3 trials registered in Clinical Trials Registry-India: A cross-sectional study
Source: PLoS One. 2023 Sep 20;18(9):e0284434. doi: 10.1371/journal.pone.0284434 (PMC10511072; doi:10.1371/journal.pone.0284434)
Supplement: Attachment — Submitted filename: Response to reviewers.doc [file pone.0284434.s011.doc]

**Our responses to the reviewers’ comments:**

Reviewer #1:

1. However, one concern that I have is that per se, having higher patients from India is not a bad thing. Authors need to make this clearer. What is their main concern with India being overrepresented in the trials and why? As long as this is done a priori with all necessary permissions from Indian authorities. Similarly, increasing participation from India later in the trial also should be OK, if the processes are followed.

[We have added the following lines to the Discussion.

“This work is concerned with the over-recruitment of participants from India in international trials. Undoubtedly trials conducted in India have undergone review by Ethics Committees. However it is known that the processing of a trial proposal by an ethics committee may be perfunctory [12]. All trials in India have also received regulatory permission. Here, too, it is known that permission may have been granted in a casual manner [13]. And, as mentioned earlier, both Indian and foreign researchers have commented on the ethical transgressions that have taken place in some trials run in India. For the 62 completed trials most of the trials with 60% or more planned or actual recruitment had foreign sponsors. Likewise, for the 362 trials that were listed as not completed, most of the cases with 60% or more planned recruitment had a foreign sponsor. Given the history of exploitation of trial participants in India, and given that all trials carry an element of risk, we believe that there is an unfair burden-benefit ratio on Indian recruitees if foreign sponsors, that are conducting trials in multiple countries, recruit too many patients from this country.”

We have commented on burden-benefit ratio in our response to Comment 5.]

- - - - - - - - - - - - - - - - - - - - - - - - - - - - - - - - - - - - - - - - - -

2. Today Indian companies want to make drug for the world and generally licensing in the home state is a requirement. So, what is wrong if they make medicines for conditions which are not “preferentially prevalent in India”.

Our (slightly revised) sentence is as follows: “Eight trials were sponsored by non-Western companies located in Brazil, Malaysia or Russia. The remaining 10 studies were sponsored by companies based in Australia, Austria, Italy, Switzerland, or the US. The latter 18 studies related to conditions that are not preferentially prevalent in India. As such 18 trials (5% of 362) had foreign sponsors that were potentially problematic.” So, we are not judging what Indian companies should or should not do. We are only saying that if a condition is not preferentially present in India, then there is no reason for a foreign sponsor to have very high recruitment from India. Let the foreign sponsors recruit participants from multiple countries, and keep the recruitment from India at less than 60%. (Please also refer to our justification of 60%, that is now added in the beginning of the Discussion.)

To illustrate why we object, we provide (for the reviewer, not in the manuscript) two quotes:

1. A quote about how trials come to India (and other lower/middle income countries) in an exploitative way: “AstraZeneca explains that the reasons for conducting placebo controlled trials with schizophrenic patients exclusively in low- and middle incomes countries is because “almost all Western ethics committees do not approve this kind of trials anymore because of ethical concerns and therefore AstraZeneca is compelled to look for destinations outside Western Europe as these placebo controlled studies are still required by the EMEA and the FDA for market authorisation” (‘Putting Contract Research Organisations on the Radar’, Mariëtte van Huijstee & Irene Schipper, 2011).

2. “We provide an example of ethics dumping in three trials conducted from 1998 to 2015 in urban and rural India on testing for cervical cancer... These trials have been condemned as unethical by public health experts and ethicists as the participants were not offered the same level of protection and consideration as participants in high income countries would have been.” (Schroeder D, Cook Lucas J, Fenet S, Hirsch F (eds) (2016) “Ethics Dumping” – Paradigmatic Case Studies, a report for TRUST)

- - - - - - - - - - - - - - - - - - - - - - - - - - - - - - - - - - - - - - - - - -

3. There are definitely problems with the CTRI website and its updation. Are these problems only for multinational trials OR are also seen in Indian trials. This is important before we infer malafide intentions.

**[**We completely agree that CTRI’s problems relate to all trials, not just those sponsored by multinationals. We are not inferring malafide intentions of the CTRI staff, or CDSCO. In 2019, we published a 17-page paper (Pillamarapu et al. *Trials*.) on some of the major categories of problems with CTRI and its data. Similar problems are found in public registries in other countries as well, as documented in that paper. Highlighting problems can lead to remedial action, and therefore we believe there is value in it. We quote (for the reviewer, not in the manuscript) from a PhD thesis from Oxford University, on the issue of clinical trial registries: “In the absence of statutory enforcement, open public audit is widely recognised as a valuable tool to increase accountability and improve quality.^433,434^] (DeVito, N., 2022, Trial Registries for Transparency and Accountability in Clinical Research. PhD thesis. Available at <https://ora.ox.ac.uk/objects/uuid:3c901316-5c24-4b42-a78e-1037d56d35e4>)

We have added the following line to the Conclusion: “Nor are we inferring malafide intentions of the CTRI staff, or of CDSCO.”

**- - - - - - - - - - - - - - - - - - - - - - - - - - - - - - - - - - - - - - - - - -**

Comments:

4. Title: needs rephrasing as “some” should not be cause of concern. Better to be neutral – “Representation from India in multinational trials registered in national trial registry”.

[We have changed the title to ‘Representation from India in multinational, interventional, Phase 2 or 3 trials registered in Clinical Trials Registry-India: A cross-sectional study’]

- - - - - - - - - - - - - - - - - - - - - - - - - - - - - - - - - - - - - - - - - -

5. Authors do not explain how they defined unfair burden-benefit ratio. Similarly, they need to define their main outcome variable (participation from India) and its measurement in the methods section.

[In the Discussion, we have now explained our belief that ‘60% participation from India’ is a limit to the fraction that is acceptable in terms of burden-benefit ratio. And in the Methods section we have defined the main outcome variable.]

- - - - - - - - - - - - - - - - - - - - - - - - - - - - - - - - - - - - - - - - - -

6. There is poor differentiation between methods/ results and discussion. Needs to be rewritten. Results starts with a statement – we posed three questions which is clearly a methods component.

[We were not sure how to proceed with this comment. If (as stated below in Reviewer Comment 8), Figure 1 is a part of Results and not Methods, then it is difficult to move the ‘we posed three questions’ para to the Methods since these questions arose from the finding of the two sets of data (earlier named Set A and Set B and now named the ‘Completed Set’, and the ‘Not Completed Set’, respectively, in response to Reviewer 2’s request to make the naming more informative). For that reason we have not moved this para from Results to Methods. However we have moved Figure 1.]

- - - - - - - - - - - - - - - - - - - - - - - - - - - - - - - - - - - - - - - - - -

7. Issue of Sanofi Pasteur and Shanta Biotech – if at all it needs to be in the paper, it should be in discussion.

[We have moved these details to the S8 File where more details are provided about each trial.]

- - - - - - - - - - - - - - - - - - - - - - - - - - - - - - - - - - - - - - - - - -

8. For example, figure 1 is a part of result and not methods.

[We have moved Figure 1 to the results.]

- - - - - - - - - - - - - - - - - - - - - - - - - - - - - - - - - - - - - - - - - -

9. Might be better to give details of the trials (table 1) in supplement.

[Table 1 details are already available in S7 File. We have deleted Table 1.]

- - - - - - - - - - - - - - - - - - - - - - - - - - - - - - - - - - - - - - - - - -

10. There are many arbitrary definitions and cut-offs which need to be justified.

[In the Discussion, we have now addressed the issue of (a) 60% as an acceptable cut off for recruitment from India; (b) the categories of what could or could not be considered an acceptable increase in the fraction of recruitment from India, and (c) for trials without a ‘Date of Study Completion’, the various assumptions made in order to calculate whether or not a trial is likely to have been completed.]

- - - - - - - - - - - - - - - - - - - - - - - - - - - - - - - - - - - - - - - - - -

11. 60% is an example. 60% or more actual recruitment – was this 60% out of total or 60% more than the planned (denominator is the planned number).

[It is 60% of the total. We have now clarified what the denominator is for various percentages. throughout the manuscript.]

- - - - - - - - - - - - - - - - - - - - - - - - - - - - - - - - - - - - - - - - - -

12. I would not put not studying before 2013 as a limitation.

[We have removed this limitation.]

- - - - - - - - - - - - - - - - - - - - - - - - - - - - - - - - - - - - - - - - - -

13. Figure 1. Should be like a typical flow charts and explain exclusions as well. It would be useful if data on all phases of trials are provided before picking phase 2-3 trials.

[Figure 1 has been redone, with the numbers for, and descriptions of, all the trials that were excluded.]

- - - - - - - - - - - - - - - - - - - - - - - - - - - - - - - - - - - - - - - - - -

Reviewer #2:

14. The paper needs a structured abstract, and the raw numbers in the abstract would benefit from percentages.

[a. Regarding the structured abstract, in the ‘Instructions to authors’, the relevant points about the Abstract are:

• Describe the main objective(s) of the study

• Explain how the study was done, including any model organisms used, without methodological detail

• Summarize the most important results and their significance.

There is no mention of sub-headings in an Abstract, and when we checked a few recent papers on the PLOS ONE site, most were in the format of a single paragraph. We have been in a dilemma as to what to do, and have kept the current format.]

[b. We have included percentages both in the Abstract and in the main text.]

- - - - - - - - - - - - - - - - - - - - - - - - - - - - - - - - - - - - - - - - - -

15. The title might need to give a clearer sense of the types of trials included and should probably give a sense of the study design for the interested reader.

[We have changed the title to ‘Representation from India in multinational, interventional, Phase 2 or 3 trials registered in Clinical Trials Registry-India: A cross-sectional study’]

- - - - - - - - - - - - - - - - - - - - - - - - - - - - - - - - - - - - - - - - - -

16. I would move the reason for the eight year period to the methods, as I felt that justification for that was only introduced late in the paper.

[We have done this.]

- - - - - - - - - - - - - - - - - - - - - - - - - - - - - - - - - - - - - - - - - -

17. What was an ‘ambiguous’ trial (page 5). Some examples might be useful here as there are lots of these and they are not really considered further in the analysis.

[We have now provided 3 examples of the types of ambiguities in the Results section.

All 384 cases are detailed in S6 File.xls. Based on this comment by Reviewer 2, we revisited S6 File, and subdivided the 384 cases into multiple sheets based on the type of ambiguity, as follows.

|  | **Category of problem** | **Number of trials** |
| --- | --- | --- |
| 1. | Entirely foreign trials. | 22 |
| 2. | Terminated in one or both fields, ‘Recruitment Status (Global)’ or ‘Recruitment Status (India)’. | 77 |
| 3. | ‘Recruitment Status (India)’ was ‘Suspended’. | 1 |
| 4. | ‘Total Sample Size’ and ‘Sample Size from India’ are the same, and therefore they appear to be Indian trials. However information in either ‘Recruitment Status_Global’ or ‘Date of first enrollment_Global’ is inconsistent with an Indian trial. | 256 |
| 5. | ‘Total Sample Size’ exceeds ‘Sample Size from India’ and therefore they appear to be multinational trials. However for ‘Recruitment Status_Global’, all the trials say ‘Not applicable’. | 18 |
| 6. | Other trials | 10 |

Based on this data, we have reworded the sentences as follows.

**Original sentences**: Based on these criteria, we obtained 5580 Indian studies, 473 multinational ones and 384 ambiguous cases (S6 File).

**New sentences**: BBased on these criteria, we obtained 5580 Indian studies, 473 multinational ones and 384 other trials (S6 File). Of the 384 other cases, 22 were entirely foreign trials; 77 were terminated either in India or elsewhere; 1 was suspended in India; 256 appeared to be Indian trials based on data in some fields, but had ambiguous information in other fields; 18 appeared to be multinational trials based on data in some fields, but had ambiguous information in other fields; and 10 trials had other kinds of discrepancies. We provide three examples from these 384 cases in Table 1.**]**

**- - - - - - - - - - - - - - - - - - - - - - - - - - - - - - - - - - - - - - - - - -**

The study is largely desccriptive and I think the methods are sensible and appropriate, with the main issues around the exact procedures used to assign trials to various categories.

- - - - - - - - - - - - - - - - - - - - - - - - - - - - - - - - - - - - - - - - - -

18. I do think the reason for the 60% criterion needs justification. As they highlight, it may be too low, but they need to give a sense of the reasons behind that threshold, as it is not clear why that was chosen.

We have added the following para to the Discussion:

[Trials come with risks for the participants. They also come with potential (a) direct benefit to the participants, or (b) indirect benefit to those of the same ethnicity in case the intervention is subsequently approved and is affordable and accessible to similar populations. In a multinational trial, where there is at least one country other than India, there ought to be a fair distribution of both the risks and potential benefits to the participants in the participating countries. As such, the fraction of participants from India should never be more than 50%, and proportionately lower based on how many countries participate in the trial. However, given that India has almost 1.4 billion people, it is almost a certainty that there will be more patients with a given condition or disease in India than in any other country, except perhaps China. Logistically speaking, it ought to be easier to conduct a trial in fewer countries, provided there are enough patients. So, in the interest of each trial being conducted in a realistic time-frame, and without the extra costs of setting up sites in too many countries, we believe that recruiting 60% of the participants from India is ethical. ]

- - - - - - - - - - - - - - - - - - - - - - - - - - - - - - - - - - - - - - - - - -

19. There is a good argument for some sensitivity analyses around this figure, since it is so important. To avoid confusing the main message, they might be included as an ‘additional file’ for interested readers to show how different thresholds impact on the results.

[This has been done, and suitable information added in the main manuscript.]

- - - - - - - - - - - - - - - - - - - - - - - - - - - - - - - - - - - - - - - - - -

20. Labelling (and naming) studies as ‘problematic’ or ‘ethically questionable’ may be an issue. I assume all these studies have received ethical clearance of some form, and although I do not feel that means that they cannot be reconsidered in terms of ethical issues by other observers, it is quite a leap to apply such labels, without the authors/companies having right of reply. Over-recruitment in particular settings does raise ethical issues, but I do not think it is by definition ‘unethical’ or ‘questionable’ or ‘unacceptable’ (which are quite loaded terms), and I think that is an important distinction. I would use less emotive language in labelling. The ethical issues can be highlighted in the introduction and discussion.

[We deleted Table 1 in response to Reviewer Comment 9. We have removed the ‘Comment’ section from Table 3. Ethical issues are now mentioned only in the Introduction and Discussion.]

- - - - - - - - - - - - - - - - - - - - - - - - - - - - - - - - - - - - - - - - - -

21. The labelling of studies as Set A and Set B is not very helpful, and it would be much better to have succinct but meaningful labels for these based on the actual criteria used to define them.

[Set A is now the ‘Completed Set’, and Set B is the ‘Not Completed Set’]

- - - - - - - - - - - - - - - - - - - - - - - - - - - - - - - - - - - - - - - - - -
