# Supplementary material for: Representation from India in multinational, interventional, phase 2 or 3 trials registered in Clinical Trials Registry-India: A cross-sectional study
Source: PLoS One. 2023 Sep 20;18(9):e0284434. doi: 10.1371/journal.pone.0284434 (PMC10511072; doi:10.1371/journal.pone.0284434)
Supplement: Attachment — Submitted filename: Response to reviewers.doc [file pone.0284434.s012.doc]

**Point-by-point response to the Editor’s and the reviewers’ comments.**

- - - - - -- - - - - - - - - - - - - - -

**Editor's comments:**

The Editor’s comments were a re-iteration of some of Reviewer #1’s comments. As such, all of them have been responded to below.

- - - - - -- - - - - - - - - - - - - - -

**Reviewers' comments:**

Reviewer #1:

1. While I share the concerns of the authors, I would prefer to adopt a non-judgemental approach to writing the paper and focus on providing objective view. They could voice their concerns in the discussion but not throughout the paper.

[In the first round of revisions, Reviewer 2 had commented “The ethical issues can be highlighted in the introduction and discussion.”, which we did. We do need to mention our concerns in the Introduction, since they form the rationale for the study. As such, this comment by Reviewer 1 has already been addressed.]
- - - - - -- - - - - - - - - - - - - - -

2. My second concern is that the authors say that their prime concern is the Indian representation, while the major section of the paper is on information available in CTRI. Either they broaden the focus on information on International trials in CTRI portal and define clear indicators for its evaluation. Right now it is a hotch-potch.

[We have analyzed Indian representation, from data available in CTRI. These records have data fields for both Indian participation and ‘global participation’. For certain analyses, we have used the fields linked to Indian participation and for some global participation. There is nothing hotch-potch about our handling of the data. We are quite used to handling CTRI data, as evidenced by the following publications from our group: Pillamarapu et al, 2019 (doi: [10.1186/S13063-019-3592-0](https://trialsjournal.biomedcentral.com/articles/10.1186/s13063-019-3592-0)), Kumari et al, 2020 (doi: [10.1371/journal.pone.0234925](https://journals.plos.org/plosone/article/peerReview?id=10.1371/journal.pone.0234925)), Chakraborty and Saberwal, 2022 (doi: 10.20529/IJME.2022.033), Chakraborty et al, 2022 (<https://doi.org/10.1371/journal.pgph.0000617>).]

- - - - - -- - - - - - - - - - - - - - -

3, Finally the data on India representation comes from very few trials.

[Yes, and that is due to the poor quality of the data in CTRI which has been the focus of some of our published work, such as a 17-page paper, Pillamarapu et al, 2019 An analysis of deficiencies in the data of interventional drug trials registered with Clinical Trials Registry – India. (doi: [10.1186/S13063-019-3592-0](https://trialsjournal.biomedcentral.com/articles/10.1186/s13063-019-3592-0)). In the current manuscript, we have provided a flowchart that shows why the numbers have been cut down at various stages. Also, in the Conclusion, we have mentioned “Further, the records of several studies that were probably completed were not updated in CTRI in a timely manner.”, which is one of the reasons for the low numbers. We are happy to enhance the Discussion by talking about the poor quality of the records, that leads to these low numbers. We could also add the low numbers as a Limitation.

The data is a quantitative description of a potential problem. Although observers have lamented that participants from India are over-represented (Eg. Pandey et al. Strengthening ethics in clinical research. Indian J Med Res 133, March 2011, pp 339-340. https://www.academia.edu/60714767/Strengthening_ethics_in_clinical_research), to the best of our knowledge, this is the only study that tries to quantify the problem.]

- - - - - -- - - - - - - - - - - - - - -

4. Authors need to strike a balance as not having enough Indians will also be criticized in todays world and having too much as well.

[We agree that not having enough Indians in multinational trials is a problem, and in fact we have highlighted this problem – for orphan drugs – in a recent publication (Chakraborty et al. Rare disease patients in India are rarely involved in international orphan drug trials. PLOS Global Public Health, doi:10.1371/journal.pgph.0000890, 2022). However the reasons that under-representation and over-representation are problematic are different. In the former, it is that without adequate ethnic diversity, the results may not be generalizable. In the latter, it is the worry of exploitation. In this work, we have focussed on the latter. We would be happy to clarify this point in the Discussion.]
- - - - - -- - - - - - - - - - - - - - -

5. Also I do not agree, that trials should be only for public health important diseases as this would mean rare disease are not studied and they may also be higher numbers in India.

[We have not argued along these lines. All that we are saying is that if a condition or disease is widespread in the world, then there is no ethical justification for over-recruiting from India. If a disease – such as tuberculosis or leishmaniasis – is more prevalent in India, then it is not ethically problematic to recruit a larger fraction of participants from India.]

- - - - - -- - - - - - - - - - - - - - -

6. There are four major stakeholders in this issues - trial sponsors, IEC/IRBs, Drug regulatory authorities and ICMR-CTRI. Authors need to delineate clearly where each of the them is not doing their job.

[Why do we need to do this? It’s as though we are offering an apple, but are being asked to offer an orange instead. Is that fair?

We are merely raising a red flag about possible over-recruitment from India. If the phenomenon is confirmed as a problem, then future research can look into which stakeholder is responsible. Fixing responsibility would be a major exercise, and a non-trivial one, since there is very little data in the public domain that would help with that.]

7. I would also expect them to avoid arbitrary cut-offs and just present the distribution wherever possible. Key indicators could be planned enrollment, actual enrollment (%) and ratio of them.

[How does one flag an issue unless one uses cut offs? We have used an arbitrary cut off, have clarified that it is arbitrary, and have also done a sensitivity analysis. We have provided all the data, and readers can come to their own conclusions.]

- - - - - -- - - - - - - - - - - - - - -
8. Table 1 can be deleted.

[Table 1 was created in the first round of revisions, in response to Reviewer 2’s request. We would request the Editor’s advice on whether or not to delete it, since the two reviewers are requesting contradictory actions.]

- - - - - -- - - - - - - - - - - - - - -

Reviewer #2: Thank you for the revised manuscript, which deals with the majority of the issues I raised. However, I would like two minor changes

9. (a) The justification of the 60% criterion is helpful, but I think that needs to go in the Methods. They can consider the wider issues relating to that threshold and potential criticisms in the Discussion, but I feel that this is a methodological issue and needs to come quite early in the paper, even at the cost of some mild repetition. (b) The abstract also needs to include a statement that defines the 60% threshold.

[We have implemented these suggestions. We have also made some edits in the Discussion due to moving some sentences to Methods.]

- - - - - -- - - - - - - - - - - - - - -

10. There are also still a few 'loaded' phrases which I do not think are helpful. I am happy that the label their phenomenon as 'potentially problematic' (I do think 'potentially' is required). The comment about 'unfair burden' needs rephrasing because the issue is about the burden on the population, not individual recruits.

[We have reworded a sentence each in the Abstract and Discussion, as follows.]

**Abstract**:

Four of the five (7% of 62) had a foreign sponsor, and therefore there was an unfair burden-benefit ratio on Indian recruitees.

**Changed to**: Four of the five (7% of 62) had a foreign sponsor, and therefore there was an unfair burden-benefit ratio on the Indian population.

**Discussion**:

Given the history of exploitation of trial participants in India, and given that all trials carry an element of risk, we believe that there is an unfair burden-benefit ratio on Indian recruitees if foreign sponsors, that are conducting trials in multiple countries, recruit too many patients from this country.

**Changed to**: Given the history of exploitation of trial participants in India, and given that all trials carry an element of risk, we believe that there is an unfair burden-benefit ratio on the Indian population if foreign sponsors, that are conducting trials in multiple countries, recruit too many patients from this country.

- - - - - -- - - - - - - - - - - - - - -

11. The statement about 'ethically questionable' should be removed, because all they have done is identified something worthy of further consideration, and are not in a position to make a strong ethical judgement

In the Abstract and Conclusion, we have deleted ‘Each of these is an ethically questionable practice.’
- - - - - -- - - - - - - - - - - - - - -
12. I also think the reference to 'guinea pigs' on page 4 should be rephrased.
**Original line**: All of this has led to apprehensions that people in developing countries have sometimes been used as guinea pigs for trials sponsored by organizations in the developed world [6–9].

**Changed to**: All of this has led to apprehensions that people in developing countries have sometimes been exploited in trials sponsored by organizations in the developed world [6–9].

- - - - - -- - - - - - - - - - - - - - -
