# Supplementary material for: Representation from India in multinational, interventional, phase 2 or 3 trials registered in Clinical Trials Registry-India: A cross-sectional study
Source: PLoS One. 2023 Sep 20;18(9):e0284434. doi: 10.1371/journal.pone.0284434 (PMC10511072; doi:10.1371/journal.pone.0284434)
Supplement: Attachment — Submitted filename: Response to reviewers.doc [file pone.0284434.s013.doc]

ACADEMIC EDITOR:

Make sure to address the concerns of the third reviewer.

**[Authors: We have done that, as detailed below.]**

- - - - - -- - - - - - - - - - - - - - -

Reviewer #3: 

1. Introduction: The objective of the study should be re-written, so it can help the reader to know the novelty of the study.

**[Authors: We have done that, both in the Abstract and in the Introduction.]**

- - - - - -- - - - - - - - - - - - - - -
2. Some references were too old, I suggest the author update these references to the latest years. There were also several references which analyzed the Clinical Trials Registry, I think these references will help the authors. Such as: PMID: 33042806; PMID: 30863312; PMID: 35881593

**[Authors: We have carefully looked into the oldest references that we have cited. We list the 3 that are before 2010 (arbitrary cut off), below. Each of them is a highly cited article, with 227, 395 and 822 citations each. We therefore respectfully request that we may retain the 1^st^ and 3^rd^ reference. Since one of the suggested references is a suitable replacement for the 2^nd^, we have substituted it.]**

1. Nundy S, Gulhati CM. A New Colonialism? — Conducting Clinical Trials in India. N Engl J Med. **2005**;352(16): 1633–1636. doi:10.1056/NEJMp048361

[**This has been cited 227 times.**]

2. Thiers FA, Sinskey AJ, Berndt ER. Trends in the globalization of clinical trials. Nat Rev Drug Discov. **2008**;7: 13–14. doi:10.1038/nrd2441

[**This has been cited 395 times.**]

3. Glickman SW, Cairns CB, Schulman KA. Ethical and Scientific Implications of the Globalization of Clinical Research. N Engl J Med. **2009**;360(8): 816–23. doi: 10.1056/NEJMsb0803929

[**This has been cited 822 times.**]

**We have also included two of the three recommended references, as detailed below:**

PMID: 33042806: Dong J, Geng Y, Lu D, Li B, Tian L, Lin D, Zhang Y. Clinical Trials for Artificial Intelligence in Cancer Diagnosis: A Cross-Sectional Study of Registered Trials in ClinicalTrials.gov. Front Oncol. 2020 Sep 15;10:1629. doi: 10.3389/fonc.2020.01629. [**We could not determine a link between our manuscript and this reference, and therefore have not included it.**]

PMID: 30863312: Chen L, Su Y, Quan L, Zhang Y, Du L. Clinical Trials Focusing on Drug Control and Prevention of Ventilator-Associated Pneumonia: A Comprehensive Analysis of Trials Registered on ClinicalTrials.gov. Front Pharmacol. 2019 Feb 26;9:1574. doi: 10.3389/fphar.2018.01574 [**We have included this reference.**]

PMID: 35881593: Brøgger-Mikkelsen M, Zibert JR, Andersen AD, Lassen U, Hædersdal M, Ali Z, Thomsen SF. Changes in key recruitment performance metrics from 2008-2019 in industry-sponsored phase III clinical trials registered at ClinicalTrials.gov. PLoS One. 2022 Jul 26;17(7):e0271819. doi: 10.1371/journal.pone.0271819 [**We have included this reference.**]
